# Lactic Acid Bacteria—Ensuring a Safe, Healthy Food Supply for Humankind since the Dawn of Our Civilization

**DOI:** 10.3390/foods11111579

**Published:** 2022-05-27

**Authors:** Maria L. N. E. Dapkevicius

**Affiliations:** 1 Faculty of Agricultural and Environmental Sciences, University of the Azores, 9700-042 Angra do Heroísmo, Portugal; maria.ln.dapkevicius@uac.pt; 2 Institute of Agricultural and Environmental Research and Technology (IITAA), University of the Azores, 9700-042 Angra do Heroísmo, Portugal

## 1. Introduction

**Figure 1:**
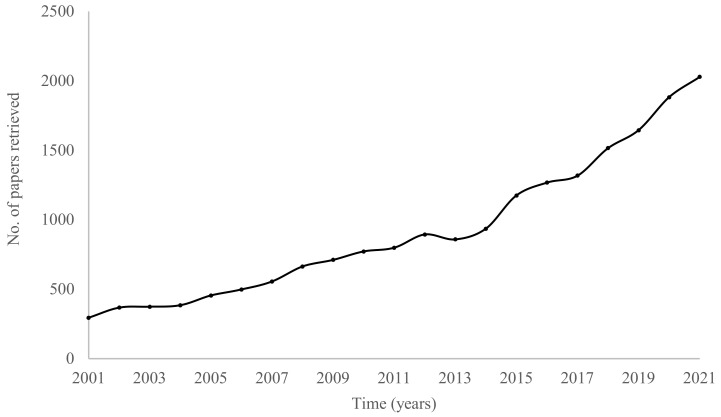
Numbers of papers retrieved in a search on the Web of Science using the terms “lactic acid bacteria” and “foods”.

Lactic acid bacteria (LAB) are part of the microbiota that inhabit several environmental niches, including foods and the gastrointestinal tract of animals and humans. The fermentation of foods with LAB has provided humankind with a low-cost, sustainable preservation methodology and has afforded us interesting flavors and textures since the dawn of our history as a species. LAB-fermented products also represent an important source of allochthonous bacteria for the human gut [1,2]. In the gut, research has shown that the metabolic activities of LAB may promote the health of their human host [2]. Thus, there has been an increase in interest for fermented foods among consumers, as demonstrated by the worldwide rise in the consumption of fermented dairy products [3]. LAB from foods have also attracted considerable interest among the research community, with a sustained growth in the numbers of papers published on this theme over the last two decades (Figure 1).

In spite of this effort, a great number of questions regarding LAB, their activities in our foods, and their roles in the human host remain unanswered, and the research on this ubiquitous group of bacteria is still lacking. LAB fermentations can provide a much-needed avenue for innovation in the food industry, but this requires a solid, science-based approach.

The research presented in this publication focuses on innovative uses of LAB fermentations sourced from traditional techniques that have been underexplored in mainstream industry. Studies on the fermentation of both dairy (yogurt) [4] and non-dairy (meats, sausages, bell peppers, and several plant-based fermented drinks) [5,6,7,8,9,10] products by lactobacilli were presented, highlighting the preservative potential, the antimicrobial properties, the impact on the product’s antioxidant activity and the overall probiotic potential associated with this group of bacteria. The hefty trove of research on the effects of LAB on olive fermentation and on human health is summarized in two review papers [11,12], aimed at presenting the state-of-the-art in these areas.

Much has been written on LAB, but much more remains to be investigated, discussed, and summarized to help us better understand—and make better use of—this exciting group of bacteria. In a world where food security is far from being achieved, LAB have an important role to play in assuring that all humans receive a safe, healthy food supply.
